# Association between a functional interleukin 6 receptor genetic variant and risk of depression and psychosis in a population-based birth cohort

**DOI:** 10.1016/j.bbi.2017.11.020

**Published:** 2018-03

**Authors:** Golam M. Khandaker, Stanley Zammit, Stephen Burgess, Glyn Lewis, Peter B. Jones

**Affiliations:** a Department of Psychiatry, University of Cambridge, Cambridge, UK; b Cambridgeshire and Peterborough NHS Foundation Trust, Cambridge, UK; c Centre for Mental Health, Addiction and Suicide Research, School of Social and Community Medicine, University of Bristol, Bristol, UK; d Institute of Psychological Medicine and Clinical Neurosciences, MRC Centre for Neuropsychiatric Genetics and Genomics, Cardiff University, Cardiff, UK; e Department of Public Health and Primary Care, University of Cambridge, Cambridge, UK; f Division of Psychiatry, University College London, London, UK

**Keywords:** ALSPAC, Avon Longitudinal Study of Parents and Children Birth Cohort, CHD, Coronary Heart Disease, CIS-R, Clinical Interview Schedule Revised, CRP, C-Reactive Protein, ELISA, Enzyme-linked Immunosorbent Assay, EPDS, Edinburgh Postnatal Depression Scale, GWAS, Genome Wide Association Study, ICD-10, International Classification of Diseases, 10th revision, IL-6, Interleukin 6, IL6R, Interleukin 6 Receptor, *IL6R*, Interleukin 6 Receptor Gene, IQ, Intelligence Quotient, PLIKSi, Psychosis-Like Symptom Interview, RCT, Randomised Controlled Trial, SCAN, Schedules for Clinical Assessment in Neuropsychiatry, SDQ, Strengths and Difficulties Questionnaire, SNP, Single Nucleotide Polymorphism, WISC III, Wechsler Intelligence Scale for Children, 3rd edition, Interleukin 6, IL-6, Interleukin 6 receptor, IL6R, Interleukin 6 receptor gene, *IL6R*, Asp358Ala, rs2228145, Inflammation, Depression, Psychosis, Mendelian randomization, Immunopsychiatry, ALSPAC birth cohort

## Abstract

**Objective:**

Interleukin 6 (IL-6) levels are commonly elevated in patients with depression and psychosis and in people who are at risk of developing these disorders. A common, functional variant in the IL6R gene (*IL6R* Asp358Ala; rs2228145 A > C) is known to dampen down inflammation by impairing IL6R signaling. We have examined the association of Asp358Ala with diagnosis of depression and psychosis, serum IL-6, CRP levels, and a number of risk factors commonly linked with inflammation, depression or psychosis. We predicted that if IL-6 were related to depression/psychosis risk causally, rather than due to confounding, Asp358Ala would be associated with risk of these disorders, serum IL-6, CRP levels, but not with any of the confounders.

**Method:**

We used data from the population-based ALSPAC birth cohort. Serum IL-6 and CRP levels were measured at age 9 years. Psychotic disorder, ICD-10 diagnosis of severe depressive episode, and total depression score were assessed at age 18 years. *IL6R* Asp358Ala was genotyped using the Illumina HumanHap550 quad genome-wide SNP genotyping platform. Risk factors assessed include sex, body mass index, social class, ethnicity, maternal education, birth weight, gestational age, maternal post-natal depression, childhood psychological and behavioral problems, and total IQ score.

**Results:**

Asp358Ala was associated with decreased risk of severe depression and/or psychosis; adjusted odds ratio for those with CC, compared with AA, genotype was 0.38 (95% CI, 0.15–0.94). The variant was associated with increased serum IL-6 levels (*P* = 5.5 × 10^−22^) but decreased serum CRP levels (*P* = 3.5 × 10^−5^), consistent with an anti-inflammatory effect downstream of IL-6. Asp358Ala was not associated with total depression score. Asp358Ala was not associated with any of the other risk factors commonly linked with inflammation, depression or psychosis (all *P* > 0.20).

**Conclusions:**

The findings provide further evidence that the IL-6/IL6R pathways are involved in pathogenesis of severe depression and psychosis, and may be novel therapeutic targets. Previously reported associations between IL-6, depression and psychosis are unlikely to be fully explained by confounding. Based on a small number of cases, findings from the current study need replication in other samples.

## Highlights

• Functional *IL6R* variant Asp358Ala (*IL6R* rs2228145; A > C) is associated with decreased risk of severe depression and/or psychosis.
• The variant exerts anti-inflammatory effect downstream of IL-6.
• rs2228145 is associated with increased serum IL-6 but decreased serum CRP levels.
• rs2228145 is not associated with common confounders of IL-6, depression and psychosis relationship.

## 1 Introduction

Experimental, clinical and epidemiological studies indicate that inflammatory cytokines may contribute to pathogenesis of depression and psychosis, of which interleukin 6 (IL-6) is one of the leading candidates. Injecting mice with IL-6, which controls serotonin transporter levels and consequently serotonin reuptake (Kong et al., 2015), leads to depression-like behaviour (Sukoff Rizzo et al., 2012). Inhibiting circulating IL-6 with a monoclonal antibody prevents depression-like behaviour in mice following exposure to stress (Hodes et al., 2014). In healthy volunteers, inflammation-induced mood deterioration and associated changes in the subgenual cingulate activity and mesolimbic connectivity are mediated by circulating IL-6 levels (Harrison et al., 2009). In patients with depression (Dowlati et al., 2010, Goldsmith et al., 2016, Haapakoski et al., 2015, Howren et al., 2009) and psychosis (Miller et al., 2011, Miller et al., 2013, Potvin et al., 2008, Upthegrove et al., 2014) who are acutely unwell, concentrations of IL-6 and other inflammatory markers are elevated compared with controls, which tend to normalize after recovery but continue to be elevated in treatment resistant patients (Goldsmith et al., 2016, Maes et al., 1997, O'Brien et al., 2007). Treatment with a monoclonal antibody against IL-6 receptor (IL6R) may improve symptoms of depression (Kappelmann et al., 2016) and schizophrenia (Miller et al., 2016).

Epidemiological studies based on prospective cohorts suggest, higher IL-6 levels are associated with cognitive symptoms of depression (Gimeno et al., 2009) and depression severity subsequently (Khandaker et al., 2014). Our work based on a population-based prospective birth cohort indicates that higher levels of IL-6 in childhood at age 9 years are associated with increased risks of developing depressive and psychotic symptoms subsequently in early-adulthood at age 18 years in a linear, dose-response fashion (Khandaker et al., 2014). Elevated IL-6 levels in childhood are also associated with persistent depressive symptoms subsequently during the second decade of life (Khandaker et al., 2017). Confounding is an important alternative explanation for epidemiological observations. Although evidence for associations between IL-6, depression and psychosis remained after controlling for a number of potential confounders, residual confounding from unmeasured factors still might account for these associations.

We have carried out a targeted genetic association analysis to examine whether association between serum IL-6 levels, depression and psychosis are consistent with a causal role of inflammation in these disorders or whether these associations could be explained by confounding (see below). To this end, we have focused on a common, functional single nucleotide polymorphism (SNP) in the IL6R gene (*IL6R* Asp358Ala; rs2228145 A > C; formerly known as rs8192284), which is known to regulate IL-6 bioactivity. Cell-based experiments have previously shown that the minor 358Ala allele decreases inflammatory activity by reducing surface expression of IL6R on CD4 + T lymphocytes and monocytes, which results in decreased responsiveness of cells to IL-6 (Ferreira et al., 2013, Reich et al., 2007). The variant is associated with higher levels of IL-6 but lower levels of CRP, and is protective for a number of physical illnesses associated with inflammation such as coronary heart disease (CHD) (Collaboration et al., 2012, Swerdlow et al., 2012) and type-1 diabetes (Ferreira et al., 2013). The variant is also associated with decreased risk of schizophrenia (Kapelski et al., 2015). However, to our knowledge no study has examined the association between Asp358Ala and depression. Furthermore, associations between Asp358Ala and circulating IL-6 and CRP levels have been observed in adults (Collaboration et al., 2012), but we are not aware of any studies examining these associations in childhood.

Based on findings from previous studies, we hypothesized that Asp358Ala would be associated with elevated serum IL-6 but decreased serum CRP levels at age 9 years, and with decreased risks of depression and psychosis at age 18 years in the ALSPAC birth cohort. We also examined the relationship between Asp358Ala and a number of risk factors commonly linked with inflammation, depression or psychosis (e.g., age, sex, social class, ethnicity, body mass index, IQ). If the variant were associated with IL-6, CRP, depression, and psychosis, but not with the confounders, this would indicate that the variant affects psychiatric risk by altering levels of inflammation. This would also indicate that previously reported associations between IL-6, depression and psychosis are unlikely to be fully explained by confounding (see discussion). In addition to using depression as continuous measure, we examined risk of severe depression defined according to ICD-10 criteria (WHO, 1992). This is because focusing on severe illness can increase the likelihood of detecting genetic associations for depression by reducing phenotypic heterogeneity (Converge Consortium, 2015). We also calculated risk of severe depression and/or psychosis; we considered severe depression and psychosis together due to their phenotypic and aetiological overlap (see discussion), and to increase statistical power.

## 2 Materials and methods

### 2.1 Description of cohort and sample

The ALSPAC birth cohort comprises 14,062 live births from pregnant women resident in county Avon, a geographically defined region in southwest of England, with expected dates of delivery between April 1991 and December 1992 (http://www.bristol.ac.uk/alspac/). Parents completed regular postal questionnaires about all aspects of their child’s health and development from birth. Since age 7, the children attended an annual assessment clinic during which they participated in various face-to-face interviews and physical tests. Samples sizes for the associations examined vary because assessments for *IL6R* genotype, serum IL-6 concentration at 9 years, serum CRP concentration at 9 years, depression at 18 years, and psychosis at 18 years were completed on different numbers of participants. We used the maximum available data to test each association (see Tables).

### 2.2 Assessments of depression at age 18 years

The computerised version of the Clinical Interview Schedule Revised (CIS-R) was self-administered by cohort participants in assessment clinics at average age 17.8 years (SD = 0.38). The CIS-R is a widely used, standardized tool for measuring common mental disorders in large community samples (Lewis et al., 1992). In the UK, CIS-R has been used in National Psychiatric Morbidity Survey, a household survey on 10,000 individuals representative of the UK population, in 1993 and 2007 (Jenkins et al., 1997, Spiers et al., 2011). The CIS-R is a fully structured assessment, suitable for trained social survey interviewers and does not require any expert knowledge on the part of the interviewers. As such, it can also be administered using personal computers in which the subjects self-complete the questionnaire (Lewis, 1994).

The CIS-R elicits responses to 14 symptoms of depression experienced in past week, and provides a diagnosis of depression according to ICD-10 criteria. We used severe depressive episode defined according to ICD-10 criteria as the main outcome. In addition, we used CIS-R total depression score as a continuous outcome measure. Total depression score ranged from zero to 21, which was calculated by summing symptom scores for depression, depressive thoughts, fatigue, concentration, and sleep problems.

### 2.3 Assessments of psychotic disorder at age 18 years

Psychotic symptoms were identified through the face-to-face, semi-structured Psychosis-Like Symptom Interview (PLIKSi) conducted by trained psychology graduates in assessment clinics, and were coded according to the definitions and rating rules for the Schedules for Clinical Assessment in Neuropsychiatry (SCAN) (WHO, 1994). The PLIKSi has good inter-rater and test-retest reliability (both kappa = 0.8) (Zammit et al., 2013). Psychotic symptoms covering the three main domains of ‘positive’ psychotic symptoms occurring since age 12 were elicited: hallucinations (visual, auditory); delusions (spied on, persecution, thoughts read, reference, control, grandiosity, other); thought interference (insertion, withdrawal, broadcasting). After cross-questioning, interviewers rated symptoms as not present, suspected, or definitely psychotic. For suspected or definite symptoms, interviewers also recorded frequency; impact on affect, social function, educational/ occupational function; help seeking; and attributions, such as fever, hypnopompic/ hypnogogic state, or drugs. Based on these data, an operational definition of psychotic disorder was created as the presence of definite psychotic symptoms not attributable to the effects of sleep/fever, and where the symptom: (1) occurred at least once per month over the past six months, and, (2) caused severe distress, or had a very negative impact on social/occupational function, or led to help-seeking from a professional source (Zammit et al., 2013).

### 2.4 Measurement of serum IL-6 and CRP concentrations at age 9 years

Blood samples were collected at non-fasting state at average age 9.9 years (SD = 0.32), immediately spun and frozen at −80 °C. Inflammatory markers were assayed in 2008 after a median of 7.5 years in storage with no previous freeze-thaw cycles during this period. IL-6 was measured by enzyme-linked immunosorbent assay (ELISA) (R&D systems, UK), and high sensitivity CRP by automated particle-enhanced immunoturbidimetric assay (Roche UK). All inter-assay coefficients of variation were <5%. The minimum detection limit for IL-6 was 0.007 pg/mL, and that for CRP was 0.01 mg/L. This represents the lowest measureable analytic level that can be distinguished from zero. In the total sample with valid inflammatory marker data at age 9 years, IL-6 values ranged from 0.007 to 20.051 pg/mL (N = 5076), and CRP values ranged from 0.01 to 67.44 mg/L (N = 5086).

### 2.5 *IL6R* SNP selection and genotyping

The study focused on a specific functional genetic variant (Asp358Ala; rs2228145) because it is well characterised with regards to its effect on IL6R signaling. Asp358Ala impairs classic IL6R signaling, and hence, dampen inflammation by reducing membrane bound IL6R levels (Ferreira et al., 2013). Previous studies have reported associations between Asp358Ala (or variants closely linked with it) and circulating inflammatory markers (Collaboration et al., 2012, Reich et al., 2007), CHD (Collaboration et al., 2012, Swerdlow et al., 2012), and auto-immune diseases such as type-1 diabetes (Ferreira et al., 2013). In total, 9912 unselected participants from the ALSPAC birth cohort were genotyped using the Illumina HumanHap550 quad genome-wide SNP genotyping platform by 23 and Me subcontracting the Wellcome Trust Sanger Institute, Cambridge, UK and the Laboratory Corporation of America, Burlington, NC, USA. After extensive quality control, high quality genotype data were available from 8355 participants (84% of those genotyped); rs2228145 was directly genotyped on the array. The variant was in Hardy-Weinberg equilibrium (observed frequencies for the common homozygotes (AA), heterozygotes (AC), and rare homozygotes (CC) genotypes were 2892, 4000 and 1463, respectively; Chi-squared = 1.55; df = 1; *P* = 0.213). We re-examined the association of rs2228145 with severe depression and psychosis by excluding participants who were related (identity-by-descent/ IBD cut off = 0.05).

### 2.6 Assessment of potential confounders

For each variable, age of assessment and available sample size used to examine association with Asp358Ala have been presented in the results section. Briefly, birth weight, gestational age and sex were recorded at birth. Body mass index (weight in kg divided by height in meter squared) was assessed around blood collection for IL-6 assay. Age at psychiatric assessment for depression and psychosis at 18 years was recorded in months. As per the UK Office of National Statistics classification system, father’s social class was recorded in six categories: I, II, III non-manual, III manual, IV, V (in descending order with professionals and higher managerial workers representing social class I). Mother’s highest educational achievement was recorded in four groups (secondary school, vocational qualification, O level, A level, degree). Ethnicity was coded as a categorical variable with the British White group comprising 97.4% of the sample. Mother’s postnatal depression was measured by the self-report Edinburgh Postnatal Depression Scale administered at 8 weeks postpartum (Cox et al., 1987). IQ was measured by the Wechsler Intelligence Scale for Children (WISC III, 3rd UK edition) at age 8 years (Wechsler et al., 1992). Mothers completed the parent version of the Strengths and Difficulties Questionnaire (SDQ) when the study child was 7 years old. The SDQ is an age appropriate, valid and reliable tool for measuring psychological and behavioural problems in young children (Goodman, 1997).

### 2.7 Statistical analysis

We used logistic regression to examine the relationship between *IL6R* genotype and the outcomes of severe depression, psychosis, and severe depression and/or psychosis, coded as binary variable. Odds ratios (ORs) for each outcome were calculated for participants with AC and CC genotype, compared with AA genotype. The regression models were adjusted for sex, body mass index, ethnicity, father’s social class, and mother’s highest educational qualification. We used linear regression to examine the relationship between CIS-R total depression score (continuous variable) and *IL6R* genotype. In addition, we used one-way analysis of variance to compare mean total depression score among three groups defined according to *IL6R* genotype. We used independent sample Kruskal Wallis test to compare distributions of total depression scores among these groups.

The association between *IL6R* genotype and serum IL-6, CRP concentrations (natural log-transformed values) was examined using linear regression. For the associations between *IL6R* genotype and psychiatric risk factors: one-way analysis of variance and linear regression were was used for continuous variable (age, birth weight, gestational age, body mass index, mother’s Edinburgh postnatal depression score at 8 weeks postpartum, Strengths and Difficulties Questionnaire total difficulties score at 7 years, child’s total Wechsler IQ score at 8 years). Chi-squared test was used for categorical variable (sex, ethnicity, father’s social class, mother’s education).

## 3 Results

### 3.1 Association between *IL6R* genotype Asp358Ala (rs2228145 A>C), severe depression and psychosis

**Fig. 1:**
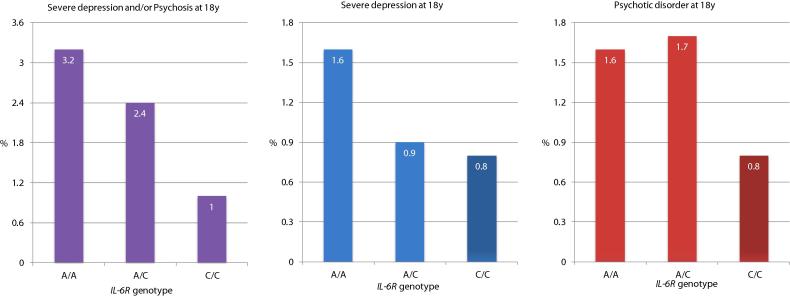
Cases of Severe Depression and Psychosis at Age 18 Years Grouped by *IL6R* Genotype Asp358Ala (rs2228145 A > C).

The minor allele frequency of Asp358Ala was 41% based on 8355 participants from the ALSPAC birth cohort. Analysis for the association between Asp358Ala and severe depression and/or psychosis at age 18 years were based on 3251 participants. The sample included 79 cases of severe depression and/or psychosis (see below for further information). Risk of severe depression and/or psychosis decreased in a linear fashion for each copy of the minor allele 358Ala; rs2228145 [C] (Fig. 1). The OR for severe depression and/or psychosis in those with the CC genotype compared with those with the AA genotype was 0.32 (95% CI, 0.13–0.76); *P-*value = 0.010, which remained statistically significant after adjusting for sex, body mass index, ethnicity, father’s social class, and mother’s highest educational level (Table 1). The OR for linear trend for association between *IL6R* genotype and severe depression and/or psychosis also remained statistically significant after adjusting for potential confounders; adjusted OR = 0.65 (95% CI, 0.44–0.95); *P-*value = 0.026. Analyses for the association of Asp358Ala with severe depression and psychosis as separate outcomes were based on 3406 and 3521 participants respectively. These analyses revealed similar results indicating a protective effect of the CC genotype although confidence intervals for the ORs became wider and included the null.

**Table 1:** The Odds Ratios for Severe Depression and/or Psychosis at Age 18 Years in the ALSPAC Birth Cohort for the *IL6R* Genotype Asp358Ala (rs2228145 A > C).

Genotype	Sample	Case, No. (%)	Odds Ratio (95% CI)	
			Unadjusted	Adjusted^1^
Severe Depression and/or Psychosis				
A/A	1127	36 (3.2)	1 [reference]	1 [reference]
A/C	1548	37 (2.4)	0.74 (0.47–1.18)	0.70 (0.41–1.20)
C/C	576	6 (1.0)	0.32 (0.13–0.76)	0.38 (0.15–0.94)
Linear trend	3251	79 (2.4)	0.63 (0.45–0.88)	0.65 (0.44–0.95)

Severe Depression				
A/A	1176	19 (1.6)	1 [reference]	1 [reference]
A/C	1621	14 (0.9)	0.53 (0.26–1.06)	0.57 (0.26–1.24)
C/C	601	5 (0.8)	0.50 (0.18–1.35)	0.59 (0.21–1.66)
Linear trend	3406	38 (1.1)	0.64 (0.40–1.05)	0.71 (0.43–1.19)

Psychosis				
A/A	1220	20 (1.6)	1 [reference]	1 [reference]
A/C	1669	29 (1.7)	1.06 (0.59–1.88)	1.09 (0.55–2.17)
C/C	632	5 (0.8)	0.47 (0.18–1.28)	0.38 (0.11–1.35)
Linear trend	3521	54 (1.5)	0.79 (0.53–1.17)	0.75 (0.47–1.20)

1 ORs have been adjusted for sex, body mass index, ethnicity, father’s social class, and mother’s highest education.

Out of 3251 participants with data on *IL6R* genotype and diagnosis of severe depression and/or psychosis, 173 were related (IBD cut off = 0.05). Sensitivity analysis after removing these participants showed results similar to the main analysis. The OR for severe depression and/or psychosis in those with the CC genotype compared with those with the AA genotype was 0.34 (95% CI, 0.14–0.82); *P-*value = 0.016. Evidence for this association attenuated after adjusting for sex, body mass index, ethnicity, father’s social class, and mother’s highest educational level quite possibly due to missing data for some these confounders; adjusted OR = 0.42 (95% CI, 0.17–1.04); *P-*value = 0.062 (Online Supplementary Table 1). However, the adjusted OR for linear trend for association between *IL6R* genotype and severe depression and/or psychosis remained statistically significant after adjusting for potential confounders; adjusted OR = 0.66 (95% CI, 0.45–0.99); *P-*value = 0.046. Additional sensitivity analyses using severe depression and psychosis as separate outcomes also showed similar results as the main analysis (Online Supplementary Tables 2 and 3).

In our dataset, out of 79 cases of severe depression and/or psychosis, 43 participants met criteria for psychosis only (i.e., no co-morbid severe depression) and 30 met criteria for severe depression only (i.e. no co-morbid psychosis), and 6 met criteria for both severe depression and psychosis. We explored the relationship of *IL6R* genotype with diagnosis of severe depression only and psychotic disorder only. Cross-tabulation of rs2228145 genotype by diagnosis showed that the CC genotype was protective for both diagnoses (Online Supplementary Tables 4 and 5). This is in line with our main analysis presented in Table 1 that examined severe depression and psychosis as separate outcomes but ignored comorbidity. In the group with both severe depression and psychosis (N = 6), the percentage of participants with AA, AC and CC genotype was the same (Online Supplementary Table 6).

### 3.2 Association between Asp358Ala and CIS-R depression total score

Data on CIS-R depression total score at age 18 years and *IL6R* genotype were available for 3400 participants. Mean depression score tended to decrease for each copy of the minor 358Ala [C] allele (Table 2); however, difference in mean depression scores among groups with AA, AC and CC genotype was not statistically significant. Similarly, distributions of depression scores among these groups were not significantly different. Linear regression did not find evidence for an association between Asp358Ala and total depression score at age 18 years (coefficient −0.114; SE = 0.097; *P =* .243).

**Table 2:** CIS-R Depression Total Score at Age 18 Years for the *IL6R* Genotype Asp358Ala (rs2228145 A > C).

*IL6R* Genotype	Sample	Depression Score, Mean (SD)	Test Statistic; *P*-value^1^	Depression Score, Median (IQR)	Test Statistic; *P*-value^2^
A/A	1174	3.33 (4.15)	F = 0.748; df = 2; *P* = 0.473	2 (0–5)	F = 0.672; df = 2; *P* = 0.715
A/C	1619	3.26 (3.95)		2 (0–5)	
C/C	607	3.09 (3.82)		2 (0–5)	

1 One-way analysis of variance was used to compare mean depression score among three groups.

2 Independent Sample Kruskal Wallis test was used to compare distributions of depression scores among three groups.

### 3.3 Association between Asp358Ala, serum IL-6 and CRP levels and other risk factors

**Fig. 2:**
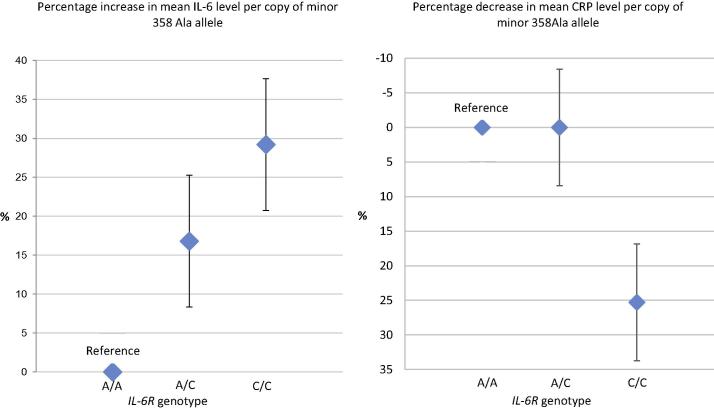
Change in Serum IL-6 and CRP Concentrations at Age 9 Years per Copy of the Minor 358Ala Allele.

Asp358Ala was strongly associated with serum IL-6 (β = 0.182; SE = 0.019; *P* = 5.5 × 10^−22^) and CRP concentrations at age 9 years (β = –0.110; SE = 0.027; *P* = 3.5 × 10^−5^). For each copy of the minor 358Ala allele serum IL-6 concentration increased by about 15% (Fig. 2; Table 3). Carriers of the homozygous minor CC genotype had lower CRP compared with carriers of the homozygous AA genotype, although this was not statistically significant. Asp358Ala was not associated with any of the other risk factors commonly linked with inflammation, depression or psychosis; all *P >* 0.20 (Table 4). We carried out additional analysis using linear regression to test the association of Asp358Ala with risk factors that were recorded as continuous variables; no evidence for association was found; all *P* > 0.10 (Online Supplementary Table 7).

**Table 3:** Concentrations of Serum IL-6 and CRP at Age 9 years by the *IL6R* genotype Asp358Ala (rs2228145 A > C).

Inflammatory Marker	*IL6R* Genotype	Sample	Mean (SD)	F-statistic; *P-*value^1^
IL-6 (pg/ml)				
	A/A	1437	1.13 (1.42)	F = 11.53; *P =* 1.1 × 10^−5^
	A/C	2032	1.32 (1.69)^2^	
	C/C	748	1.46 (1.62)^2^	
	Total	4217	1.28 (1.60)	

CRP (mg/L)				
	A/A	1442	0.83 (2.95)	F = 1.83; *P* = 0.15
	A/C	2037	0.84 (2.70)	
	C/C	748	0.62 (2.55)^3^	
	Total	4227	0.80 (2.76)	

1 One-way Analysis of Variance was used to compare mean concentrations of IL-6 and CRP among groups with the AA, AC and CC genotype.

2 Calculation of increase in serum IL-6 mean concentration for one copy of C allele: compared with the AA group, mean IL-6 in the AC group was 0.19 pg/ml higher (i.e., 16.8% increase from 1.13 pg/ml for one copy of the C allele). Compared with the AA group, mean IL-6 in the CC group was 0.33 pg/ml higher (i.e., 29.2% increase from 1.13 pg/ml for two copy of the C allele). Therefore, average increase in mean IL-6 concentration for one copy of C allele is (16.8 + 29.2) ÷ 3 = 15.33%.

3 Calculation of decrease in mean CRP concentration in the group with CC, compared with AA, genotype: 0.83–0.62 = 0.21; (0.21 ÷ 0.83) × 100 = 25.3%.

**Table 4:** Association between *IL6R* Genotype Asp358Ala (rs2228145 A > C), Serum IL-6 and CRP Concentrations, and Other Risk Factors.

Risk Factor^1^	Age of Assessment	Available Sample	Statistic for Association^2^	*P-*value for Association
Inflammatory Markers				
Serum IL-6 Level	Mean 9.9y (SD = 0.32)	4217	0.182 (0.019)	5.5 × 10^−22^
Serum CRP Level	Mean 9.9y (SD = 0.32)	4227	−0.110 (0.027)	3.5 × 10^−5^

Other Risk Factors				
Age at Diagnosis of Depression and Psychosis	Mean 17.8y (SD = 0.38)	3535	1.557	0.211
Sex	At birth	8228	1.113^2^	0.573
Ethnicity	At birth	7372	1.146^2^	0.564
Father’s Social Class	At birth	6746	9.568^2^	0.654
Mother’s Highest Education	At birth	7398	6.144^2^	0.631
Birth Weight	At birth	7767	0.892	0.410
Gestational Age	At birth	7864	0.679	0.507
Mother’s Postnatal Depression	8-week post-partum	7156	0.057	0.944
Childhood Behavioural Problems	Mean 6.8y (SD = 0.11)	5718	0.555	0.574
Childhood Intelligence	Mean 8.7y (SD = 0.32)	5509	0.353	0.702
Body Mass Index	Mean 9.9y (SD = 0.32)	5813	1.52	0.218

1 Linear regression was used for IL-6 and CRP (natural logarithm transformed values); One Way Analysis of Variance was used for Age, birth weight, gestational age, Body Mass Index, mother’s Edinburgh Postnatal Depression Score at 8 weeks postpartum, Strengths and Difficulties Questionnaire total difficulties score at 7 years, child’s total Wechsler IQ score at 8 years.

2 Chi-squared test was used for sex, ethnicity, father’s social class, mother’s highest education.

## 4 Discussion

Using data from a population-based birth cohort, we report that a common, functional variant in the IL6R gene (*IL6R* Asp358Ala; rs2228145 A > C) that is known to dampen down inflammation is associated with decreased risk of severe depression and/or psychosis. The variant is associated with increased serum IL-6 levels but decreased serum CRP levels, which is consistent with an anti-inflammatory effect of the minor 358Ala allele downstream of IL-6 previously reported by others (Ferreira et al., 2013). Asp358Ala was not associated with any of the risk factors commonly linked with inflammation, depression or psychosis, such as age, sex, body mass, social class, ethnicity, maternal post-natal depression, and IQ. Taken together, these findings indicate that the variant affects risk of major psychiatric disorder (severe depression and/or psychosis) by altering levels of inflammation. Previously reported associations between IL-6, depression and psychosis are unlikely to be fully explained by confounders commonly linked with inflammation, depression or psychosis. The variant was not associated with total depression score, which indicates that the IL6R pathway may be relevant for severe rather than mild depression. Based on a small number of cases the findings need replication in other samples, but provide further evidence that the IL-6/IL6R pathways are involved in pathogenesis of severe depression and psychosis.

We have focused on a specific genetic variant (Asp358Ala; rs2228145) because it is well characterised with regards to its effect on IL6R signaling (Ferreira et al., 2013, Reich et al., 2007). The findings are consistent with biological actions of this genetic variant and with previous epidemiological studies reporting a protective effect of this variant for a number of physical illnesses including CHD (Collaboration et al., 2012), atrial fibrillation (Schnabel et al., 2011), abdominal aortic aneurysm (Harrison et al., 2013), and type-1 diabetes (Ferreira et al., 2013). Asp358Ala is known to dampen down inflammation by reducing membrane bound IL-6 signaling. Although 358Ala increases transcription of the soluble IL6R isoform and not the membrane-bound isoform, 358Ala reduces surface expression of IL-6R on CD4 + T lymphocytes and monocytes (up to 28% reduction per allele). Importantly, reduced expression of membrane-bound IL-6R results in impaired IL-6 responsiveness, as measured by decreased phosphorylation of the transcription factors STAT3 and STAT1 following stimulation with IL-6 (Ferreira et al., 2013). A large collaborative *meta*-analysis of 82 studies has previously reported that Asp358Ala is associated with increased levels of circulating IL-6 and sIL6R, but decreased levels of CRP in adults (Collaboration et al., 2012). We report that the variant is associated with increased IL-6 but decreased CRP levels assessed in childhood, and is protective for severe depression and/or psychosis assessed in early adulthood.

The analytic approach is informed by Mendelian randomization, which posits that if a biomarker is causally related to a disease, genetic variant(s) controlling activity of that biomarker should also be associated with the disease (Burgess et al., 2015, Davey Smith and Ebrahim, 2003). Using genetic variants as proxy for exposure overcomes confounding because genetic variants are inherited at random during meiosis, so are unrelated to potential confounders (measured or unmeasured). Previous research has provided observational evidence for an association between IL-6, depression and psychosis using population-based longitudinal (Gimeno et al., 2009, Khandaker et al., 2014) and cross-sectional studies (Dowlati et al., 2010, Goldsmith et al., 2016, Haapakoski et al., 2015, Howren et al., 2009, Miller et al., 2011, Potvin et al., 2008, Upthegrove et al., 2014). We show that Asp358Ala is strongly associated with serum concentrations of IL-6 and CRP, but not with any of the risk factors commonly linked with inflammation, depression or psychosis. These findings indicate confounding is not a complete explanation for previously observed associations between IL-6, depression and psychosis.

The selection of a specific well-characterized genetic variant is useful because it can serve as a proxy for drug actions. Epidemiological genetic findings can be checked against a drug, so can directly inform development of new treatments. Previous studies of cardiovascular disease have used a similar genetic variant specific approach comparing effects of Asp358Ala with tocilizumab (Collaboration et al., 2012, Swerdlow et al., 2012), and SNPs in *IL-1R* locus with anakinra (IL-1R antagonist) (Interleukin 1 Genetics Consortium, 2015). Tocilizumab is a humanized monoclonal antibody against the IL6R which inhibits both IL-6 classic and trans signaling (Calabrese and Rose-John, 2014). Improvement in depressive symptoms after treatment with tocilizumab in clinical trials (Gossec et al., 2015, Kappelmann et al., 2016, Traki et al., 2014) is consistent with the observed protective effect of Asp358Ala for severe depression in our epidemiological sample.

The variant was not associated with total depression score, which indicates that the IL6R pathway may be relevant for severe rather than mild depression. An alternative explanation might be age of assessment for depression and psychosis. Our sample was relatively young (average age at assessment of outcomes 18 years). A recent GAWS has reported that earlier-onset major depressive disorder is genetically more similar to schizophrenia and bipolar disorder than adult-onset major depression (Power et al., 2017). Although results for severe depression and psychosis were similar, indicating a protective effect of CC genotype, the ORs were not statistically significant. We observed a significant association with Asp358Ala when severe depression and/or psychosis was used as outcome. We combined severe depression and psychosis to increase statistical power, and due to considerable overlap between depression and psychosis in clinical presentation (APA, 2013), environmental and genetic risk factors (Green et al., 2010, Smith et al., 2016).

To our knowledge, this is one of the first studies to examine the association of Asp358Ala with depression, circulating inflammatory markers and other psychiatric risk factors using the same sample. Published literature on association between Asp358Ala and schizophrenia is mixed. It has been reported that the CC, compared with AA genotype, may decrease (Kapelski et al., 2015) or increase (Hudson and Miller, 2016) risk of schizophrenia, while one study did not find an association (Sun et al., 2008). A search of existing GWAS results from the Psychiatric Genomics Consortium revealed that Asp358Ala is associated with risks of schizophrenia (*P =* 0.019) (Schizophrenia Working Group of the Psychiatric Genomics, 2014), attention deficit hyperactivity disorder (*P =* 0.008) (Neale et al., 2010), but not with major depressive disorder (*P =* 0.954) (Major Depressive Disorder Working Group of the Psychiatric et al., 2013). The relatively young age of the sample and choice of outcome (severe depression and/or psychosis), might explain difference with findings from previous studies.

Limitations of the work include a relatively small number of cases of severe depression and psychosis in the ALSPAC cohort. However, the cases were phenotypically well defined and were of similar age, which might have aided detection of an association with the genetic variant. Relatively young age of the sample precludes generalizability of findings to older adults. In future, studies with larger samples and longer follow-up are required. Due to the small number of cases, we have not examined associations between Asp358Ala, depression and psychosis in male and female participants separately. However, the association between Asp358Ala and severe depression and/or psychosis remained significant after controlling for sex. We have not carried out cell-based functional experiments to examine the effects of Asp358Ala on IL6R signaling in our participants. However, association of the genetic variant (minor 358Ala allele) with decreased serum CRP levels are consistent with an anti-inflammatory effect of this genotype downstream of IL-6 as demonstrated by others previously (Ferreira et al., 2013). We have not carried out a two-stage least squared regression analysis to obtain a Mendelian randomization causal estimate for serum IL-6. Although Asp358Ala is associated with serum IL-6 and soluble IL6R levels, it is not clear whether the effect of Asp358Ala on risks of severe depression and/or psychosis is mediated by specifically serum IL-6, soluble IL6R or membrane-bound IL6R.

A potential role for IL-6 in pathogenesis of depression, psychosis and other major mental disorders is supported by experimental and clinical studies (Dantzer, 2004, Dantzer et al., 2008, Khandaker and Dantzer, 2016, Miller et al., 2009, Raison et al., 2006). Depression-like behaviour is observed in mice following experimental immune-activation with injection of lipopolysaccharide in peripheral circulation (O'Connor et al., 2009) or direct intracerebroventricular injection of IL-6 (Sukoff Rizzo et al., 2012). IL-6 influences serotonin transporter levels and consequently serotonin reuptake (Kong et al., 2015). Peripheral IL-6 plays a key role in mediating the effect of stress on brain as demonstrated by experiments in mice (Hodes et al., 2014) and in human volunteers (Harrison et al., 2009). In mice, inhibiting IL-6 with a monoclonal antibody prevents depression-like behaviour following exposures to stress (Hodes et al., 2014). Non-specific peripheral immune activation caused by injection of lipopolysaccharide in healthy volunteers increases serum IL-6 levels as well as inducing low mood, anxiety and reduced cognitive performance (Reichenberg et al., 2001). Inflammation-induced mood deterioration and associated changes in the subgenual cingulate activity and mesolimbic connectivity are mediated by circulating IL-6 (Harrison et al., 2009).

Low-grade inflammation might be a common mechanism underlying the high comorbidity between depression, schizophrenia, CHD and diabetes. Longitudinal studies have shown that higher serum concentrations of IL-6 increase future risks of heart disease (Danesh et al., 2008), diabetes mellitus (Pradhan et al., 2001), depression and psychosis (Khandaker et al., 2014). Previous studies have reported that Asp358Ala has a protective effect on heart disease (Collaboration et al., 2012, Swerdlow et al., 2012) and type-1 diabetes (Ferreira et al., 2013), which is consistent with our findings, and indicates a potentially causal role for inflammation in these illnesses.

Demonstrating a causal role for low-grade inflammation in depression and psychosis may lead to new treatments. Elevated serum inflammatory marker concentrations predict poor response to antidepressants (Yoshimura et al., 2009) and antipsychotics (Mondelli et al., 2015), while treatment resistant patients continue to show elevated cytokine levels (Goldsmith et al., 2016, Maes et al., 1997, O'Brien et al., 2007). Therefore, stratification of patients according to their immune phenotype might aid prediction of treatment response. Anti-inflammatory drugs such as non-steroidal anti-inflammatory agents (Kohler et al., 2014) and cytokine modulators (Kappelmann et al., 2016) may be helpful for some patients with depression and schizophrenia (Muller et al., 2002, Sommer et al., 2014). Recently, a proof-of-concept randomised controlled trial (RCT) of infliximab, an anti-TNF monoclonal antibody, has reported improvements in patients with treatment resistant depression characterized by high inflammation at baseline (Raison et al., 2013). Based on human population-based data our findings provide further evidence that the IL-6/IL6R pathways are involved in pathogenesis of severe depression and psychosis. The field now needs RCTs of cytokine modulators targeting IL-6/IL6R for patients with depression and psychosis, specifically those with evidence of inflammation, to translate these results into benefits for patients.

## References

[b0005] APA (2013). Diagnostic and statistical manual of mental disorders.

[b0010] Burgess S., Timpson N.J., Ebrahim S., Davey Smith G. (2015). Mendelian randomization: where are we now and where are we going?. Int. J. Epidemiol..

[b0015] Calabrese L.H., Rose-John S. (2014). IL-6 biology: implications for clinical targeting in rheumatic disease. Nat. Rev. Rheumatol..

[b0020] Collaboration I.R.G.C.E.R.F., Sarwar N., Butterworth A.S., Freitag D.F., Gregson J., Willeit P., Gorman D.N., Gao P., Saleheen D., Rendon A., Nelson C.P., Braund P.S., Hall A.S., Chasman D.I., Tybjaerg-Hansen A., Chambers J.C., Benjamin E.J., Franks P.W., Clarke R., Wilde A.A., Trip M.D., Steri M., Witteman J.C., Qi L., van der Schoot C.E., de Faire U., Erdmann J., Stringham H.M., Koenig W., Rader D.J., Melzer D., Reich D., Psaty B.M., Kleber M.E., Panagiotakos D.B., Willeit J., Wennberg P., Woodward M., Adamovic S., Rimm E.B., Meade T.W., Gillum R.F., Shaffer J.A., Hofman A., Onat A., Sundstrom J., Wassertheil-Smoller S., Mellstrom D., Gallacher J., Cushman M., Tracy R.P., Kauhanen J., Karlsson M., Salonen J.T., Wilhelmsen L., Amouyel P., Cantin B., Best L.G., Ben-Shlomo Y., Manson J.E., Davey-Smith G., de Bakker P.I., O'Donnell C.J., Wilson J.F., Wilson A.G., Assimes T.L., Jansson J.O., Ohlsson C., Tivesten A., Ljunggren O., Reilly M.P., Hamsten A., Ingelsson E., Cambien F., Hung J., Thomas G.N., Boehnke M., Schunkert H., Asselbergs F.W., Kastelein J.J., Gudnason V., Salomaa V., Harris T.B., Kooner J.S., Allin K.H., Nordestgaard B.G., Hopewell J.C., Goodall A.H., Ridker P.M., Holm H., Watkins H., Ouwehand W.H., Samani N.J., Kaptoge S., Di Angelantonio E., Harari O., Danesh J. (2012). Interleukin-6 receptor pathways in coronary heart disease: a collaborative meta-analysis of 82 studies. Lancet.

[b0025] Converge Consortium (2015). Sparse whole-genome sequencing identifies two loci for major depressive disorder. Nature.

[b0030] Cox J.L., Holden J.M., Sagovsky R. (1987). Detection of postnatal depression. Development of the 10-item Edinburgh Postnatal Depression Scale. Br. J. Psychiatr. J. Mental Sci..

[b0035] Danesh J., Kaptoge S., Mann A.G., Sarwar N., Wood A., Angleman S.B., Wensley F., Higgins J.P., Lennon L., Eiriksdottir G., Rumley A., Whincup P.H., Lowe G.D., Gudnason V. (2008). Long-term interleukin-6 levels and subsequent risk of coronary heart disease: two new prospective studies and a systematic review. PLoS Med..

[b0040] Dantzer R. (2004). Cytokine-induced sickness behaviour: a neuroimmune response to activation of innate immunity. Eur. J. Pharmacol..

[b0045] Dantzer R., O'Connor J.C., Freund G.G., Johnson R.W., Kelley K.W. (2008). From inflammation to sickness and depression: when the immune system subjugates the brain. Nat. Rev. Neurosci..

[b0050] Davey Smith G., Ebrahim S. (2003). ‘Mendelian randomization’: can genetic epidemiology contribute to understanding environmental determinants of disease?. Int. J. Epidemiol..

[b0055] Dowlati Y., Herrmann N., Swardfager W., Liu H., Sham L., Reim E.K., Lanctot K.L. (2010). A meta-analysis of cytokines in major depression. Biol. Psychiatr..

[b0060] Ferreira R.C., Freitag D.F., Cutler A.J., Howson J.M., Rainbow D.B., Smyth D.J., Kaptoge S., Clarke P., Boreham C., Coulson R.M., Pekalski M.L., Chen W.M., Onengut-Gumuscu S., Rich S.S., Butterworth A.S., Malarstig A., Danesh J., Todd J.A. (2013). Functional IL6R 358Ala allele impairs classical IL-6 receptor signaling and influences risk of diverse inflammatory diseases. PLoS Genet..

[b0065] Gimeno D., Kivimaki M., Brunner E.J., Elovainio M., De Vogli R., Steptoe A., Kumari M., Lowe G.D., Rumley A., Marmot M.G., Ferrie J.E. (2009). Associations of C-reactive protein and interleukin-6 with cognitive symptoms of depression: 12-year follow-up of the Whitehall II study. Psychol. Med..

[b0070] Goldsmith D.R., Rapaport M.H., Miller B.J. (2016). A meta-analysis of blood cytokine network alterations in psychiatric patients: comparisons between schizophrenia, bipolar disorder and depression. Mol. Psychiatr..

[b0075] Goodman R. (1997). The Strengths and Difficulties Questionnaire: a research note. J. Child Psychol. Psychiatr. Allied Disciplines.

[b0080] Gossec L., Steinberg G., Rouanet S., Combe B. (2015). Fatigue in rheumatoid arthritis: quantitative findings on the efficacy of tocilizumab and on factors associated with fatigue. The French multicentre prospective PEPS Study. Clin. Exp. Rheumatol..

[b0085] Green E.K., Grozeva D., Jones I., Jones L., Kirov G., Caesar S., Gordon-Smith K., Fraser C., Forty L., Russell E., Hamshere M.L., Moskvina V., Nikolov I., Farmer A., McGuffin P., Wellcome Trust Case, Control, C., Holmans P.A., Owen M.J., O'Donovan M.C., Craddock N. (2010). The bipolar disorder risk allele at CACNA1C also confers risk of recurrent major depression and of schizophrenia. Mol. Psychiatr..

[b0090] Haapakoski R., Mathieu J., Ebmeier K.P., Alenius H., Kivimaki M. (2015). Cumulative meta-analysis of interleukins 6 and 1beta, tumour necrosis factor alpha and C-reactive protein in patients with major depressive disorder. Brain Behav. Immun..

[b0095] Harrison N.A., Brydon L., Walker C., Gray M.A., Steptoe A., Critchley H.D. (2009). Inflammation causes mood changes through alterations in subgenual cingulate activity and mesolimbic connectivity. Biol. Psychiatr..

[b0100] Harrison S.C., Smith A.J., Jones G.T., Swerdlow D.I., Rampuri R., Bown M.J., Aneurysm C., Folkersen L., Baas A.F., de Borst G.J., Blankensteijn J.D., Price J.F., van der Graaf Y., McLachlan S., Agu O., Hofman A., Uitterlinden A.G., Franco-Cereceda A., Ruigrok Y.M., van't Hof, Powell F.N.J.T., van Rij A.M., Casas J.P., Eriksson P., Holmes M.V., Asselbergs F.W., Hingorani A.D., Humphries S.E. (2013). Interleukin-6 receptor pathways in abdominal aortic aneurysm. Eur. Heart J..

[b0105] Hodes G.E., Pfau M.L., Leboeuf M., Golden S.A., Christoffel D.J., Bregman D., Rebusi N., Heshmati M., Aleyasin H., Warren B.L., Lebonte B., Horn S., Lapidus K.A., Stelzhammer V., Wong E.H., Bahn S., Krishnan V., Bolanos-Guzman C.A., Murrough J.W., Merad M., Russo S.J. (2014). Individual differences in the peripheral immune system promote resilience versus susceptibility to social stress. PNAS.

[b0110] Howren M.B., Lamkin D.M., Suls J. (2009). Associations of depression with C-reactive protein, IL-1, and IL-6: a meta-analysis. Psychosom. Med..

[b0115] Hudson, Z.D., Miller, B.J., 2016. Meta-analysis of cytokine and chemokine genes in schizophrenia. Clin. Schizophrenia Related Psychoses. 27454212

[b0120] Interleukin 1 Genetics Consortium (2015). Cardiometabolic effects of genetic upregulation of the interleukin 1 receptor antagonist: a Mendelian randomisation analysis. The lancet. Diab. Endocrinol..

[b0130] Jenkins R., Lewis G., Bebbington P., Brugha T., Farrell M., Gill B., Meltzer H. (1997). The National Psychiatric Morbidity surveys of Great Britain–initial findings from the household survey. Psychol. Med..

[b0135] Kapelski P., Skibinska M., Maciukiewicz M., Wilkosc M., Frydecka D., Groszewska A., Narozna B., Dmitrzak-Weglarz M., Czerski P., Pawlak J., Rajewska-Rager A., Leszczynska-Rodziewicz A., Slopien A., Zaremba D., Twarowska-Hauser J. (2015). Association study of functional polymorphisms in interleukins and interleukin receptors genes: IL1A, IL1B, IL1RN, IL6, IL6R, IL10, IL10RA and TGFB1 in schizophrenia in Polish population. Schizophrenia Res..

[b0140] Kappelmann N., Lewis G., Dantzer R., Jones P.B., Khandaker G.M. (2016). Antidepressant activity of anti-cytokine treatment: a systematic review and meta-analysis of clinical trials of chronic inflammatory conditions. Mol. Psychiatr..

[b0145] Khandaker G.M., Dantzer R. (2016). Is there a role for immune-to-brain communication in schizophrenia?. Psychopharmacology.

[b0150] Khandaker G.M., Pearson R.M., Zammit S., Lewis G., Jones P.B. (2014). Association of serum interleukin 6 and C-reactive protein in childhood with depression and psychosis in young adult life: a population-based longitudinal study. JAMA Psychiatr..

[b0155] Khandaker G.M., Stochl J., Zammit S., Goodyer I.M., Lewis G., Jones P.B. (2017). Childhood inflammatory markers and intelligence as predictors of subsequent persistent depressive symptoms: a longitudinal cohort study. Psychol. Med..

[b0160] Kohler O., Benros M.E., Nordentoft M., Farkouh M.E., Iyengar R.L., Mors O., Krogh J. (2014). Effect of anti-inflammatory treatment on depression, depressive symptoms, and adverse effects: a systematic review and meta-analysis of randomized clinical trials. JAMA Psychiatr..

[b0165] Kong E., Sucic S., Monje F.J., Savalli G., Diao W., Khan D., Ronovsky M., Cabatic M., Koban F., Freissmuth M., Pollak D.D. (2015). STAT3 controls IL6-dependent regulation of serotonin transporter function and depression-like behavior. Sci. Rep..

[b0170] Lewis G. (1994). Assessing psychiatric disorder with a human interviewer or a computer. J. Epidemiol. Commun. Health.

[b0175] Lewis G., Pelosi A.J., Araya R., Dunn G. (1992). Measuring psychiatric disorder in the community: a standardized assessment for use by lay interviewers. Psychol. Med..

[b0180] Maes M., Bosmans E., De Jongh R., Kenis G., Vandoolaeghe E., Neels H. (1997). Increased serum IL-6 and IL-1 receptor antagonist concentrations in major depression and treatment resistant depression. Cytokine.

[b0185] Major Depressive Disorder Working Group of the Psychiatric, G.C., Ripke, S., Wray, N.R., Lewis, C.M., Hamilton, S.P., Weissman, M.M., Breen, G., Byrne, E.M., Blackwood, D.H., Boomsma, D.I., Cichon, S., Heath, A.C., Holsboer, F., Lucae, S., Madden, P.A., Martin, N.G., McGuffin, P., Muglia, P., Noethen, M.M., Penninx, B.P., Pergadia, M.L., Potash, J.B., Rietschel, M., Lin, D., Muller-Myhsok, B., Shi, J., Steinberg, S., Grabe, H.J., Lichtenstein, P., Magnusson, P., Perlis, R.H., Preisig, M., Smoller, J.W., Stefansson, K., Uher, R., Kutalik, Z., Tansey, K.E., Teumer, A., Viktorin, A., Barnes, M.R., Bettecken, T., Binder, E.B., Breuer, R., Castro, V.M., Churchill, S.E., Coryell, W.H., Craddock, N., Craig, I.W., Czamara, D., De Geus, E.J., Degenhardt, F., Farmer, A.E., Fava, M., Frank, J., Gainer, V.S., Gallagher, P.J., Gordon, S.D., Goryachev, S., Gross, M., Guipponi, M., Henders, A.K., Herms, S., Hickie, I.B., Hoefels, S., Hoogendijk, W., Hottenga, J.J., Iosifescu, D.V., Ising, M., Jones, I., Jones, L., Jung-Ying, T., Knowles, J.A., Kohane, I.S., Kohli, M.A., Korszun, A., Landen, M., Lawson, W.B., Lewis, G., Macintyre, D., Maier, W., Mattheisen, M., McGrath, P.J., McIntosh, A., McLean, A., Middeldorp, C.M., Middleton, L., Montgomery, G.M., Murphy, S.N., Nauck, M., Nolen, W.A., Nyholt, D.R., O'Donovan, M., Oskarsson, H., Pedersen, N., Scheftner, W.A., Schulz, A., Schulze, T.G., Shyn, S.I., Sigurdsson, E., Slager, S.L., Smit, J.H., Stefansson, H., Steffens, M., Thorgeirsson, T., Tozzi, F., Treutlein, J., Uhr, M., van den Oord, E.J., Van Grootheest, G., Volzke, H., Weilburg, J.B., Willemsen, G., Zitman, F.G., Neale, B., Daly, M., Levinson, D.F., Sullivan, P.F., 2013. A mega-analysis of genome-wide association studies for major depressive disorder. Mol. Psychiatr. 18, 497–511. 10.1038/mp.2012.21 PMC3837431 22472876

[b0190] Miller A.H., Maletic V., Raison C.L. (2009). Inflammation and its discontents: the role of cytokines in the pathophysiology of major depression. Biol. Psychiatr..

[b0195] Miller B.J., Buckley P., Seabolt W., Mellor A., Kirkpatrick B. (2011). Meta-analysis of cytokine alterations in schizophrenia: clinical status and antipsychotic effects. Biol. Psychiatr..

[b0200] Miller B.J., Culpepper N., Rapaport M.H. (2013). C-Reactive protein levels in schizophrenia. Clin. Schizophrenia Related Psychoses.

[b0205] Miller B.J., Dias J.K., Lemos H.P., Buckley P.F. (2016). An open-label, pilot trial of adjunctive tocilizumab in schizophrenia. J. Clin. Psychiatr..

[b0210] Mondelli V., Ciufolini S., Belvederi Murri M., Bonaccorso S., Di Forti M., Giordano A., Marques T.R., Zunszain P.A., Morgan C., Murray R.M., Pariante C.M., Dazzan P. (2015). Cortisol and inflammatory biomarkers predict poor treatment response in first episode psychosis. Schizophrenia Bull..

[b0215] Muller N., Riedel M., Scheppach C., Brandstatter B., Sokullu S., Krampe K., Ulmschneider M., Engel R.R., Moller H.J., Schwarz M.J. (2002). Beneficial antipsychotic effects of celecoxib add-on therapy compared to risperidone alone in schizophrenia. Am. J. Psychiatr..

[b0220] Neale B.M., Medland S.E., Ripke S., Asherson P., Franke B., Lesch K.P., Faraone S.V., Nguyen T.T., Schafer H., Holmans P., Daly M., Steinhausen H.C., Freitag C., Reif A., Renner T.J., Romanos M., Romanos J., Walitza S., Warnke A., Meyer J., Palmason H., Buitelaar J., Vasquez A.A., Lambregts-Rommelse N., Gill M., Anney R.J., Langely K., O'Donovan M., Williams N., Owen M., Thapar A., Kent L., Sergeant J., Roeyers H., Mick E., Biederman J., Doyle A., Smalley S., Loo S., Hakonarson H., Elia J., Todorov A., Miranda A., Mulas F., Ebstein R.P., Rothenberger A., Banaschewski T., Oades R.D., Sonuga-Barke E., McGough J., Nisenbaum L., Middleton F., Hu X., Nelson S., Psychiatric G.C.A.S. (2010). Meta-analysis of genome-wide association studies of attention-deficit/hyperactivity disorder. J. Am. Acad. Child Adolesc. Psychiatry.

[b0225] O'Brien S.M., Scully P., Fitzgerald P., Scott L.V., Dinan T.G. (2007). Plasma cytokine profiles in depressed patients who fail to respond to selective serotonin reuptake inhibitor therapy. J. Psychiatr. Res..

[b0230] O'Connor J.C., Lawson M.A., Andre C., Moreau M., Lestage J., Castanon N., Kelley K.W., Dantzer R. (2009). Lipopolysaccharide-induced depressive-like behavior is mediated by indoleamine 2,3-dioxygenase activation in mice. Mol. Psychiatry.

[b0235] Potvin S., Stip E., Sepehry A.A., Gendron A., Bah R., Kouassi E. (2008). Inflammatory cytokine alterations in schizophrenia: a systematic quantitative review. Biol. Psychiatry.

[b0240] Power, R.A., Tansey, K.E., Buttenschon, H.N., Cohen-Woods, S., Bigdeli, T., Hall, L.S., Kutalik, Z., Lee, S.H., Ripke, S., Steinberg, S., Teumer, A., Viktorin, A., Wray, N.R., Arolt, V., Baune, B.T., Boomsma, D.I., Borglum, A.D., Byrne, E.M., Castelao, E., Craddock, N., Craig, I.W., Dannlowski, U., Deary, I.J., Degenhardt, F., Forstner, A.J., Gordon, S.D., Grabe, H.J., Grove, J., Hamilton, S.P., Hayward, C., Heath, A.C., Hocking, L.J., Homuth, G., Hottenga, J.J., Kloiber, S., Krogh, J., Landen, M., Lang, M., Levinson, D.F., Lichtenstein, P., Lucae, S., MacIntyre, D.J., Madden, P., Magnusson, P.K., Martin, N.G., McIntosh, A.M., Middeldorp, C.M., Milaneschi, Y., Montgomery, G.W., Mors, O., Muller-Myhsok, B., Nyholt, D.R., Oskarsson, H., Owen, M.J., Padmanabhan, S., Penninx, B.W., Pergadia, M.L., Porteous, D.J., Potash, J.B., Preisig, M., Rivera, M., Shi, J., Shyn, S.I., Sigurdsson, E., Smit, J.H., Smith, B.H., Stefansson, H., Stefansson, K., Strohmaier, J., Sullivan, P.F., Thomson, P., Thorgeirsson, T.E., Van der Auwera, S., Weissman, M.M., Converge Consortium, C.C.G.C., Breen, G., Lewis, C.M., 2017. Genome-wide association for major depression through age at onset stratification: major depressive disorder working group of the psychiatric genomics consortium Biol. Psychiatry 81, 325–335. 10.1016/j.biopsych.2016.05.010 PMC5262436 27519822

[b0245] Pradhan A.D., Manson J.E., Rifai N., Buring J.E., Ridker P.M. (2001). C-reactive protein, interleukin 6, and risk of developing type 2 diabetes mellitus. JAMA J. Am. Med. Assoc..

[b0250] Raison C.L., Capuron L., Miller A.H. (2006). Cytokines sing the blues: inflammation and the pathogenesis of depression. Trends Immunol..

[b0255] Raison C.L., Rutherford R.E., Woolwine B.J., Shuo C., Schettler P., Drake D.F., Haroon E., Miller A.H. (2013). A randomized controlled trial of the tumor necrosis factor antagonist infliximab for treatment-resistant depression: the role of baseline inflammatory biomarkers. JAMA Psychiatry.

[b0260] Reich D., Patterson N., Ramesh V., De Jager P.L., McDonald G.J., Tandon A., Choy E., Hu D., Tamraz B., Pawlikowska L., Wassel-Fyr C., Huntsman S., Waliszewska A., Rossin E., Li R., Garcia M., Reiner A., Ferrell R., Cummings S., Kwok P.Y., Harris T., Zmuda J.M., Ziv E., Health A., Body Composition S. (2007). Admixture mapping of an allele affecting interleukin 6 soluble receptor and interleukin 6 levels. Am. J. Human Genet..

[b0265] Reichenberg A., Yirmiya R., Schuld A., Kraus T., Haack M., Morag A., Pollmacher T. (2001). Cytokine-associated emotional and cognitive disturbances in humans. Arch. Gen. Psychiatry.

[b0270] Schizophrenia Working Group of the Psychiatric Genomics, C. (2014). Biological insights from 108 schizophrenia-associated genetic loci. Nature.

[b0275] Schnabel R.B., Kerr K.F., Lubitz S.A., Alkylbekova E.L., Marcus G.M., Sinner M.F., Magnani J.W., Wolf P.A., Deo R., Lloyd-Jones D.M., Lunetta K.L., Mehra R., Levy D., Fox E.R., Arking D.E., Mosley T.H., Muller-Nurasyid M., Young T.R., Wichmann H.E., Seshadri S., Farlow D.N., Rotter J.I., Soliman E.Z., Glazer N.L., Wilson J.G., Breteler M.M., Sotoodehnia N., Newton-Cheh C., Kaab S., Ellinor P.T., Alonso A., Benjamin E.J., Heckbert S.R., Candidate Gene Association Resource Atrial Fibrillation/Electrocardiography Working, G. (2011). Large-scale candidate gene analysis in whites and African Americans identifies IL6R polymorphism in relation to atrial fibrillation: the National Heart, Lung, and Blood Institute's Candidate Gene Association Resource (CARe) project. Circulation Cardiovas. Genet..

[b0280] Smith D.J., Escott-Price V., Davies G., Bailey M.E., Colodro-Conde L., Ward J., Vedernikov A., Marioni R., Cullen B., Lyall D., Hagenaars S.P., Liewald D.C., Luciano M., Gale C.R., Ritchie S.J., Hayward C., Nicholl B., Bulik-Sullivan B., Adams M., Couvy-Duchesne B., Graham N., Mackay D., Evans J., Smith B.H., Porteous D.J., Medland S.E., Martin N.G., Holmans P., McIntosh A.M., Pell J.P., Deary I.J., O'Donovan M.C. (2016). Genome-wide analysis of over 106 000 individuals identifies 9 neuroticism-associated loci. Mol. Psychiatry.

[b0285] Sommer I.E., van Westrhenen R., Begemann M.J., de Witte L.D., Leucht S., Kahn R.S. (2014). Efficacy of anti-inflammatory agents to improve symptoms in patients with schizophrenia: an update. Schizophrenia Bull..

[b0290] Spiers N., Bebbington P., McManus S., Brugha T.S., Jenkins R., Meltzer H. (2011). Age and birth cohort differences in the prevalence of common mental disorder in England: National Psychiatric Morbidity Surveys 1993–2007. Br. J. Psychiatry J. Mental Sci..

[b0295] Sukoff Rizzo S.J., Neal S.J., Hughes Z.A., Beyna M., Rosenzweig-Lipson S., Moss S.J., Brandon N.J. (2012). Evidence for sustained elevation of IL-6 in the CNS as a key contributor of depressive-like phenotypes. Transl. Psychiatry.

[b0300] Sun S., Wang F., Wei J., Cao L.Y., Qi L.Y., Xiu M.H., Chen S., Li X.H., Kosten T.A., Kosten T.R., Zhang X.Y. (2008). Association between interleukin-6 receptor polymorphism and patients with schizophrenia. Schizophrenia Res..

[b0125] Swerdlow D.I., Holmes M.V., Kuchenbaecker K.B., Engmann J.E., Shah T., Sofat R., Guo Y., Chung C., Peasey A., Pfister R., Mooijaart S.P., Ireland H.A., Leusink M., Langenberg C., Li K.W., Palmen J., Howard P., Cooper J.A., Drenos F., Hardy J., Nalls M.A., Li Y.R., Lowe G., Stewart M., Bielinski S.J., Peto J., Timpson N.J., Gallacher J., Dunlop M., Houlston R., Tomlinson I., Tzoulaki I., Luan J., Boer J.M., Forouhi N.G., Onland-Moret N.C., van der Schouw Y.T., Schnabel R.B., Hubacek J.A., Kubinova R., Baceviciene M., Tamosiunas A., Pajak A., Topor-Madry R., Malyutina S., Baldassarre D., Sennblad B., Tremoli E., de Faire U., Ferrucci L., Bandenelli S., Tanaka T., Meschia J.F., Singleton A., Navis G., Mateo Leach I., Bakker S.J., Gansevoort R.T., Ford I., Epstein S.E., Burnett M.S., Devaney J.M., Jukema J.W., Westendorp R.G., Jan de Borst G., van der Graaf Y., de Jong P.A., Mailand-van der Zee A.H., Klungel O.H., de Boer A., Doevendans P.A., Stephens J.W., Eaton C.B., Robinson J.G., Manson J.E., Fowkes F.G., Frayling T.M., Price J.F., Whincup P.H., Morris R.W., Lawlor D.A., Smith G.D., Ben-Shlomo Y., Redline S., Lange L.A., Kumari M., Wareham N.J., Verschuren W.M., Benjamin E.J., Whittaker J.C., Hamsten A., Dudbridge F., Delaney J.A., Wong A., Kuh D., Hardy R., Castillo B.A., Connolly J.J., van der Harst P., Brunner E.J., Marmot M.G., Wassel C.L., Humphries S.E., Talmud P.J., Kivimaki M., Asselbergs F.W., Voevoda M., Bobak M., Pikhart H., Wilson J.G., Hakonarson H., Reiner A.P., Keating B.J., Sattar N., Hingorani A.D., Casas J.P., (Interleukin-6 Receptor Mendelian Randomisation Analysis (IL6R MR) Consortium) (2012). The interleukin-6 receptor as a target for prevention of coronary heart disease: a mendelian randomisation analysis. Lancet.

[b0305] Traki L., Rostom S., Tahiri L., Bahiri R., Harzy T., Abouqal R., Hajjaj-Hassouni N. (2014). Responsiveness of the EuroQol EQ-5D and Hospital Anxiety and Depression Scale (HADS) in rheumatoid arthritis patients receiving tocilizumab. Clin. Rheumatol..

[b0310] Upthegrove R., Manzanares-Teson N., Barnes N.M. (2014). Cytokine function in medication-naive first episode psychosis: a systematic review and meta-analysis. Schizophrenia Res..

[b0315] Wechsler, D., Golombok, S., Rust, J., 1992. Weschler Intelligence Scale for Children (3rd Edition) (WISC–III UK). The Psychological Corporation.

[b0320] WHO (1992). The ICD-10 Classification of Mental and Behavioural Disorder: Clinical Descriptions and Disgnostic Guidelines.

[b0325] WHO (1994). SCAN: Schedules for Clinical Assessment in Neuropsychiatry Version 2.0..

[b0330] Yoshimura R., Hori H., Ikenouchi-Sugita A., Umene-Nakano W., Ueda N., Nakamura J. (2009). Higher plasma interleukin-6 (IL-6) level is associated with SSRI- or SNRI-refractory depression. Progr. Neuro-psychopharmacol. Biol. Psychiatry.

[b0335] Zammit S., Kounali D., Cannon M., David A.S., Gunnell D., Heron J., Jones P.B., Lewis S., Sullivan S., Wolke D., Lewis G. (2013). Psychotic experiences and psychotic disorders at age 18 in relation to psychotic experiences at age 12 in a longitudinal population-based cohort study. Am. J. Psychiatry.

