# Supplementary material for: Association between a functional interleukin 6 receptor genetic variant and risk of depression and psychosis in a population-based birth cohort
Source: Brain Behav Immun. 2018 Mar;69:264–72. doi: 10.1016/j.bbi.2017.11.020 (PMC5871397; doi:10.1016/j.bbi.2017.11.020)
Supplement: Supplementary data 1 [file mmc1.docx]

**Online Supplementary Material: Khandaker *et al*. Association between a Functional Interleukin 6 Receptor Genetic Variant and Risk of Depression and Psychosis in a Population-based Birth Cohort**

**Online Supplementary Table 1: The Odds Ratios for Severe Depression and/or Psychosis at Age 18 Years in the ALSPAC Birth Cohort for the *IL6R* Genotype Asp358Ala (rs2228145 A>C)**

Sensitivity analysis after removing related participants (Identity-by-Descent cut-off = 0.05)

| **Genotype** | **Sample** | **Case, No. (%)** | **Odds Ratio (95% CI)** | | | |
| --- | --- | --- | --- | --- | --- | --- |
|  |  |  | **Unadjusted analysis (N=3078)** | **Adjusted for sex and body mass index (N=2815)** | **Adjusted for father’s social class, ethnicity, mother’s highest education (N=2700)** | **Adjusted for sex, body mass index, father’s social class, ethnicity, mother’s highest education (N=2482)** |
| A/A | 1052 | 33 (3.1) | 1 [reference] | 1 [reference] | 1 [reference] | 1 [reference] |
| A/C | 1478 | 35 (2.4) | 0.75 (0.46-1.21); p=0.240 | 0.74 (0.44-1.25); p=0.267 | 0.68 (0.40-1.17); p=0.168 | 0.70 (0.40-1.23); p=0.224 |
| C/C | 548 | 6 (1.1) | 0.34 (0.14-0.82); p=0.016 | 0.39 (0.16-0.96); p=0.042 | 0.39 (0.16-0.95); p=0.038 | 0.42 (0.17-1.04); p=0.062 |
| Linear trend | 3078 | 74 (2.4) | 0.64 (0.45-0.91); p=0.013 | 0.66 (0.46-0.97); p=0.034 | 0.64 (0.44-0.94); p=0.025 | 0.66 (0.45-0.99); p=0.046 |

**Online Supplementary Table 2: The Odds Ratios for Severe Depression at Age 18 Years in the ALSPAC Birth Cohort for the *IL6R* Genotype Asp358Ala (rs2228145 A>C)**

Sensitivity analysis after removing related participants (Identity-by-Descent cut-off = 0.05)

| **Genotype** | **Sample** | **Case, No. (%)** | **Odds Ratio (95% CI)** | | | |
| --- | --- | --- | --- | --- | --- | --- |
|  |  |  | **Unadjusted analysis (N=3078)** | **Adjusted for sex and body mass index (N=2946)** | **Adjusted for father’s social class, ethnicity, mother’s highest education (N=2822)** | **Adjusted for sex, body mass index, father’s social class, ethnicity, mother’s highest education (N=2593)** |
| A/A | 1098 | 16 (1.5) | 1 [reference] | 1 [reference] | 1 [reference] | 1 [reference] |
| A/C | 1549 | 13 (0.8) | 0.57 (0.27-1.19); p=0.137 | 0.65 (0.29-1.44); p=0.298 | 0.63 (0.28-1.40); p=0.260 | 0.63 (0.27-1.43); p=0.272 |
| C/C | 580 | 5 (0.9) | 0.58 (0.21-1.61); p=0.302 | 0.72 (0.25-2.05); p=0.548 | 0.69 (0.24-1.96); p=0.492 | 0.73 (0.25-2.10); p=0.558 |
| Linear trend | 3227 | 34 (1.1) | 0.70 (0.42-1.16); p=0.176 | 0.80 (0.47-1.35); p=0.414 | 0.78 (0.46-1.32); p=0.362 | 0.80 (0.46-1.37); p=0.423 |

**Online Supplementary Table 3: The Odds Ratios for Psychotic Disorder at Age 18 Years in the ALSPAC Birth Cohort for the *IL6R* Genotype Asp358Ala (rs2228145 A>C)**

Sensitivity analysis after removing related participants (Identity-by-Descent cut-off = 0.05)

| **Genotype** | **Sample** | **Case, No. (%)** | **Odds Ratio (95% CI)** | | | |
| --- | --- | --- | --- | --- | --- | --- |
|  |  |  | **Unadjusted (N=3332)** | **Adjusted for sex and body mass index (N=3303)** | **Adjusted for father’s social class, ethnicity, mother’s highest education (N=2911)** | **Adjusted for sex, body mass index, father’s social class, ethnicity, mother’s highest education (N=2667)** |
| A/A | 1141 | 19 (1.7) | 1 [reference] | 1 [reference] | 1 [reference] | 1 [reference] |
| A/C | 1590 | 28 (1.8) | 1.06 (0.59-1.90); p=0.849 | 1.07 (0.56-2.03); p=0.831 | 0.97 (0.49-1.91); p=0.944 | 1.07 (0.53-2.19); p=0.836 |
| C/C | 601 | 5 (0.8) | 0.49 (0.18-1.33); p=0.164 | 0.34 (0.10-1.18); p=0.092 | 0.36 (0.10-1.28); p=0.116 | 0.41 (0.11-1.46); p=0.170 |
| Linear trend | 3332 | 52 (1.6) | 0.79 (0.53-1.19); p=0.270 | 0.73 (0.47-1.14); p=0.177 | 0.73 (0.45-1.15); p=0.176 | 0.76 (0.47-1.24); p=0.276 |

**Online Supplementary Table 4: Cross-tabulation of the *IL6R* Genotype Asp358Ala (rs2228145 A>C) and Diagnosis of Psychotic Disorder Only**

(Cases with co-morbid severe depression were removed from dataset)

| **Genotype** | **Diagnosed with Psychotic Disorder Only at 18 Years** | |  |
| --- | --- | --- | --- |
|  | **No, No. (%)** | **Yes, No. (%)** | **Total, No (%)** |
| A/A | 1091 (98.4) | 18 (1.6) | 1109 (100) |
| A/C | 1511 (98.4) | 24 (1.6) | 1535 (100) |
| C/C | 570 (99.8) | 1 (0.2) | 571 (100) |
| Total | 3172 (98.7) | 43 (1.3) | 3215 (100) |

**Online Supplementary Table 5: Cross-tabulation of the *IL6R* Genotype Asp358Ala (rs2228145 A>C) and Diagnosis of Severe Depression Only**

(Cases with co-morbid psychotic disorder were removed from dataset)

| **Genotype** | **Diagnosed with Severe Depression Only at 18 Years** | |  |
| --- | --- | --- | --- |
|  | **No, No. (%)** | **Yes, No. (%)** | **Total, No (%)** |
| A/A | 1091 (98.6) | 16 (1.4) | 1107 (100) |
| A/C | 1511 (99.3) | 10 (0.7) | 1521 (100) |
| C/C | 570 (99.3) | 4 (0.7) | 574 (100) |
| Total | 3172 (99.1) | 30 (0.9) | 3202 (100) |

**Online Supplementary Table 6: Cross-tabulation of the *IL6R* Genotype Asp358Ala (rs2228145 A>C) and Diagnosis of both Psychosis and Severe Depression**

(All other cases were removed from dataset)

| **Genotype** | **Diagnosed with both Psychosis and Severe Depression at 18 Years** | |  |
| --- | --- | --- | --- |
|  | **No, No. (%)** | **Yes, No. (%)** | **Total, No (%)** |
| A/A | 1091 (99.8) | 2 (0.2) | 1093 (100) |
| A/C | 1511 (99.8) | 3 (0.2) | 1514 (100) |
| C/C | 570 (99.8) | 1 (0.2) | 571 (100) |
| Total | 3172 (99.8) | 6 (0.2) | 3178 (100) |

**Online Supplementary Table 7: Results for Linear Regression Testing Association between *IL6R* Genotype Asp358Ala (rs2228145 A>C) and Other Risk Factors**

| **Risk Factor^1^** | **Age of Assessment** | **Available Sample** | **Regression co-efficient; SE** | ***P-*value for Association** |
| --- | --- | --- | --- | --- |
| Age at Diagnosis of Depression and Psychosis | Mean 17.8y (SD=0.38) | 3535 | 0.169; 0.104 | 0.105 |
| Birth Weight | At birth | 7767 | 9.674; 8.639 | 0.263 |
| Gestational Age | At birth | 7864 | 0.026; 0.029 | 0.372 |
| Mother’s Postnatal Depression | 8-week post-partum | 7156 | 0.018; 0.078 | 0.822 |
| Childhood Behavioural Problems | Mean 6.8y (SD=0.11) | 5718 | 0.090; 0.090 | 0.316 |
| Childhood Intelligence | Mean 8.7y (SD=0.32) | 5509 | 0.264; 0.315 | 0.401 |
| Body Mass Index | Mean 9.9y (SD=0.32) | 5813 | -0.081; 0.053 | 0.126 |

^1^ Mother’s postnatal depression was measured by the Edinburgh Postnatal Depression Score at 8 weeks postpartum; childhood behavioural problems were measured by the Strengths and Difficulties Questionnaire (total difficulties score) at 7 years; total Wechsler IQ score at 8 years was used as a measure of childhood intelligence.
